# A Retrospective Study of Progression-Free and Overall Survival in Pediatric Medulloblastoma Based on Molecular Subgroup Classification: A Single-Institution Experience

**DOI:** 10.3389/fneur.2017.00198

**Published:** 2017-05-12

**Authors:** Tao Jiang, Yuqi Zhang, Junmei Wang, Jiang Du, Raynald, Xiaoguang Qiu, Ying Wang, Chunde Li

**Affiliations:** ^1^Department of Neurosurgery, Beijing Tiantan Hospital, Capital Medical University, Beijing, China; ^2^Beijing Neurosurgical Institute, Capital Medical University, Beijing, China; ^3^China National Clinical Research Center for Neurological Diseases, Beijing, China; ^4^Department of Neurosurgery, Yuquan Hospital, Medical Center, Tsinghua University, Beijing, China; ^5^Beijing Chao-Yang Hospital, Capital Medical University, Beijing, China

**Keywords:** medulloblastoma, pediatrics, molecular subgroups, prognosis, treatment

## Abstract

**Background:**

Medulloblastoma (MB) has been classified into four core subgroups according to the transcriptional profile in recent years. However, some disagreement among researchers remains regarding the prognoses and most effective treatments of the different subgroups with different age distributions.

**Objective:**

The objective of this study was to analyze MB prognosis in children population based on the classification of four molecular subgroups.

**Methods:**

From January 2011 to January 2013, 84 consecutive MB patients aged underwent tumor removal at Beijing Tiantan Hospital. A total of 55 patients who ranged in age from 4 to 18 years underwent detailed follow-up. Molecular subgrouping was performed using RT-PCR.

**Results:**

The 2-year progression-free survival (PFS) and overall survival (OS) rates for the entire cohort were 76.2 ± 5.8 and 81.8 ± 5.2%, respectively. Univariate analysis revealed that the Group 4 patients had a better survival (2-year OS, 90.6 ± 5.2%) than the SHH subgroup (*P* = 0.002) and Group 3 patients (*P* = 0.008). Only two of the 23 non-metastasized Group 4 patients relapsed, and chemotherapy did significantly affect these patients (PFS, *P* = 0.685). One out of five WNT patients had tumor relapse and died at last. Large cell/anaplastic (LC/A) histology and chemotherapy were independent risk factors in multivariate analysis.

**Conclusion:**

In our study, the non-metastasized Group 4 patients had an excellent prognosis. The SHH subgroup and Group 3 patients had worst prognoses. LC/A histology had a dismal prognosis in our cohorts, which warrants intensive treatment.

## Introduction

Medulloblastoma (MB) is the most common malignant intracranial tumor in children, accounting for 20% of all pediatric brain tumors and 40% of pediatric posterior fossa tumors (1). Multimodal treatment, including tumor resection, postsurgical craniospinal irradiation, and chemotherapy, has led to improvements in prognosis over the past three decades. Traditional risk classification (2) according to patient age, tumor residue, metastasis, and microscopic histology allows for the controlled de-escalation of treatment intensity. At most medical centers, approximately two-thirds of MB patients achieve long-term survival (3). However, treatment-related sequelae, such as meningioma and neurocognitive impairment, greatly impact the quality of life of these long-term survivors. Thus, a more accurate risk classification system is warranted to reduce treatment toxicity and to enable personalized treatment.

In recent years, knowledge about MB biology has markedly increased with the use of high-throughput transcriptomic methods. According to the current consensus (2), four main groups of MB patients can be distinguished: WNT, SHH, Group 3, and Group 4. These four distinct subgroups have different outcomes and recurrence patterns. Owing to treatment heterogeneity across hospitals, some disagreement remains regarding the prognosis of these patients and most effective treatment strategy. In the current consensus, WNT and non-metastatic Group 4 patients are considered as low-risk patients, and SHH and metastatic Group 4 patients are considered as high-risk patients (2). Only little data have been published to date regarding the prognosis of pediatric MB patients in relation to the SHH, WNT, and non-SHH/WNT molecular subgroups in Chinese patients (4). Beijing Tiantan Hospital is a tertiary referral center for central nervous system tumors located in China. MB patients at our hospital are treated using similar protocols (5–7). In this study, we aimed to investigate the subgroup-specific prognoses of consecutive pediatric cases at a single institution and to assess the effects of different treatment strategies on patients in the different molecular subgroups.

## Patients and Methods

### Patients

This study was approved by the ethics committee of Beijing Tiantan Hospital, Capital Medical University. Informed consent was obtained from all participants or their parent or legal guardian. The study protocol conformed to the ethical guidelines of the 1975 Declaration of Helsinki. The patients underwent enhanced MRI of the whole spinal column to exclude possible metastasis before they received irradiation or chemotherapy. However, postsurgical cerebrospinal fluid examination was not routinely performed. Patients were classified as high-risk patients if the tumor residue was >1.5 cm^2^ and (or) if tumor metastasis was detected. In our cohort, the M1-stage patients were grouped with the M0-stage patients.

Tumor locations were classified as lateral or central according to the radiological findings. Tumor infiltration into the fourth ventricle floor (V4 floor) was identified by assessing surgical reports. The patients were classified into three subtypes according to tumor location and infiltration into the V4 floor, as previously described (5). Location subgroup 1 included central tumors without V4 floor infiltration, location subgroup 2 consisted of central tumors with V4 floor infiltration, and location subgroup 3 included tumors located in the cerebellar hemisphere or cerebellopontine angle.

The specimens were reviewed by two independent neuropathologists according to the 2007 WHO criteria (8). The tumors were subdivided into three subtypes: classic histology medulloblastoma (CMB), desmoplastic/nodular medulloblastoma (DNMB) and medulloblastoma with extensive nodularity (MBEN), and large cell/anaplastic (LC/A) MB.

All patients received postsurgical cerebrospinal irradiation (CSI) no more than 1 month after tumor removal. The patients received a CSI dose of 30.6–36.0 Gy, followed by a posterior fossa boost (23–26 Gy). Patients without metastatic tumor received CSI dose of 30.6 Gy, while patients with metastatic tumor received dose of 36.0 Gy. The patients with postsurgical cerebellar mutism or tumor metastasis received two cycles of chemotherapy before CSI. Seven out of 55 patients in our cohort did not receive immediate irradiation because of moderate-to-severe mutism. These seven mutism patients received two cycles of chemotherapy followed by CSI.

Maintenance chemotherapy included eight cycles of carboplatin, vincristine, and lomustine for the average-risk patients. In addition, the “HIT-SKK-2000/GPOH” treatment strategy (9), which included two cycles of induction chemotherapy (cyclophosphamide, vincristine, carboplatin, and etoposide), was applied to the high-risk patients. After radiotherapy, the high-risk patients received six cycles of maintenance chemotherapy, similar to the average-risk patients. Because most of the patients at our hospital received a presurgical ventriculoperitoneal shunt, intraventricular methotrexate was rarely administered. In China, chemotherapy is not covered by insurance for many patients. In our study, only patients who could afford the expense received maintenance chemotherapy.

### Molecular Diagnosis

Molecular subgroups were determined by real-time RT-PCR, as described by Kunder et al (10). Tumor tissues were obtained with the approval of the institutional review board. Fresh tumor tissues were collected during surgery, snap frozen in liquid nitrogen, and then stored at −80°C. Total RNA was extracted from frozen tissues, and spectrophotometry was performed to assess the quality and quantity. RNA (1–2 µg) was reverse transcribed using random hexamer primers and M-MLV reverse transcriptase (Invitrogen). The real-time PCR primers were designed (Table S1 in Supplementary Material) and listed as Kunder et al (10). Gene expression was assessed by SYBR Green PCR amplification using an Applied Biosystems 7900HT real-time PCR system. To determine the relative quantity of each protein-coding gene, the $2^{-\Delta \Delta {\text{C}}_{\text{T}}}$ method was used to calculate the fold change of each gene, and GAPDH was used as an internal control. All samples were assessed in triplicate, and the final data were presented as the mean of at least three individual experiments.

### Statistical Analysis

Descriptive statistics were reported in terms of absolute frequencies and percentages for the qualitative data. Follow-up was censored in January 2016. Progression-free survival (PFS) and overall survival (OS) were measured from the date of tumor resection and were calculated using the Kaplan–Meier method. PFS was calculated from the date of surgery to the date of first relapse at any site, death, or the last follow-up. OS was measured from the date of tumor resection to the date of final follow-up or death. Differences between groups were assessed using the log-rank test. All tests were two tailed, and a *P* value of ≤0.05 was considered as statistically significant. Analyses were carried out with SPSS software 17.0 (IBM, Armonk, NY, USA). The prognostic effects of gender, tumor location, histology, tumor residue, tumor metastasis, chemotherapy, and molecular subgroup were assessed. Receiver operating characteristic curves were generated with GraphPad Prism software (version 2.0). The Cox regression model was used to conduct multivariate analysis. Estimated hazard ratios were provided with 95% confidence intervals, and the *P* values were calculated using the likelihood ratio test. The variables included in analyses are mentioned earlier. Forward stepwise selection was chosen (inclusion criterion: score test, *P* ≤ 0.05; exclusion criterion: likelihood ratio test, *P* ≥ 0.10).

## Results

### Patient Characteristics

**Figure 1:**
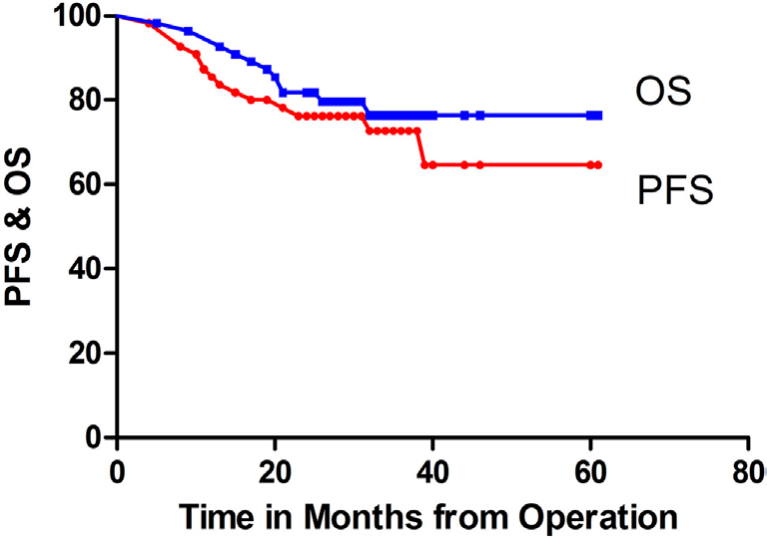
**Progression-free survival (PFS) and overall survival (OS) rates of all patients**.

A total of 55 patients who ranged in age from 4 to 18 years, with a median age of 7.0 years, underwent detailed follow-up. The patients’ clinical details are summarized in Table 1. The male/female ratio was 35:20. The follow-up times ranged from 5 to 61 months (mean, 30.2 months; median, 29.0 months). The two-year PFS and OS rates for the entire cohort were 76.2 ± 5.8 and 81.8 ± 5.2%, respectively (Figure 1).

**Table 1:** **Clinical characteristic of patients aged 3–18 years in Beijing Tiantan Hospital**.

Characteristic	No. of patients
**Age (years)**	
Median, range	7.0 (4–18)
**Gender**	
Male	35 (63.6%)
Female	20 (36.4%)
Follow-up time (months)	30.2 (5-61)
**Pathological histology**	
Classic histology medulloblastoma	37 (67.3%)
Desmoplastic/nodular medulloblastoma and medulloblastoma with extensive nodularity	12 (21.8%)
Large cell/anaplastic histology	6 (10.9%)
**Chang stage**	
M0	41 (74.5%)
M+	14 (25.5%)
**Extent of tumor removal**	
GTR	49 (89.1%)
STR	6 (10.9%)
**Chemotherapy**	
Yes	22 (40%)
No	33 (60%)
**Tumor location**	
Midline without fourth ventricle floor (V4 floor) infiltration	26 (47.3%)
Midline with V4 floor infiltration	23 (41.8%)
Lateral location	6 (10.9%)
**Molecular classification**	
WNT	5 (9.1%)
SHH	6 (10.9%)
Group 3	12 (21.8%)
Group 4	32 (58.2%)

The average-risk group included 38 patients, and the high-risk group included 17 patients, with 2-year PFS rates of 78.8 ± 6.7 and 70.1 ± 11.2%, respectively; this difference was not significant (*P* = 0.376). In addition, the 2-year PFS was 75.3 ± 6.2% for the 49 patients without tumor residue compared with 83.3 ± 15.2% for the six patients with STR (*P* = 0.534).

### Clinicopathological Prognosis in Each Molecular Subgroup

**Figure 2:**
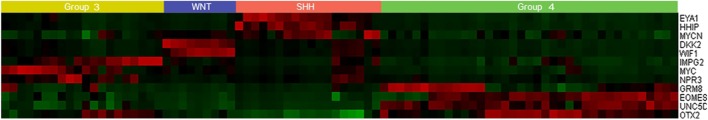
**Heat map showing differential expression of 12 protein-coding genes in 84 frozen tumor tissues**.

**Figure 3:**
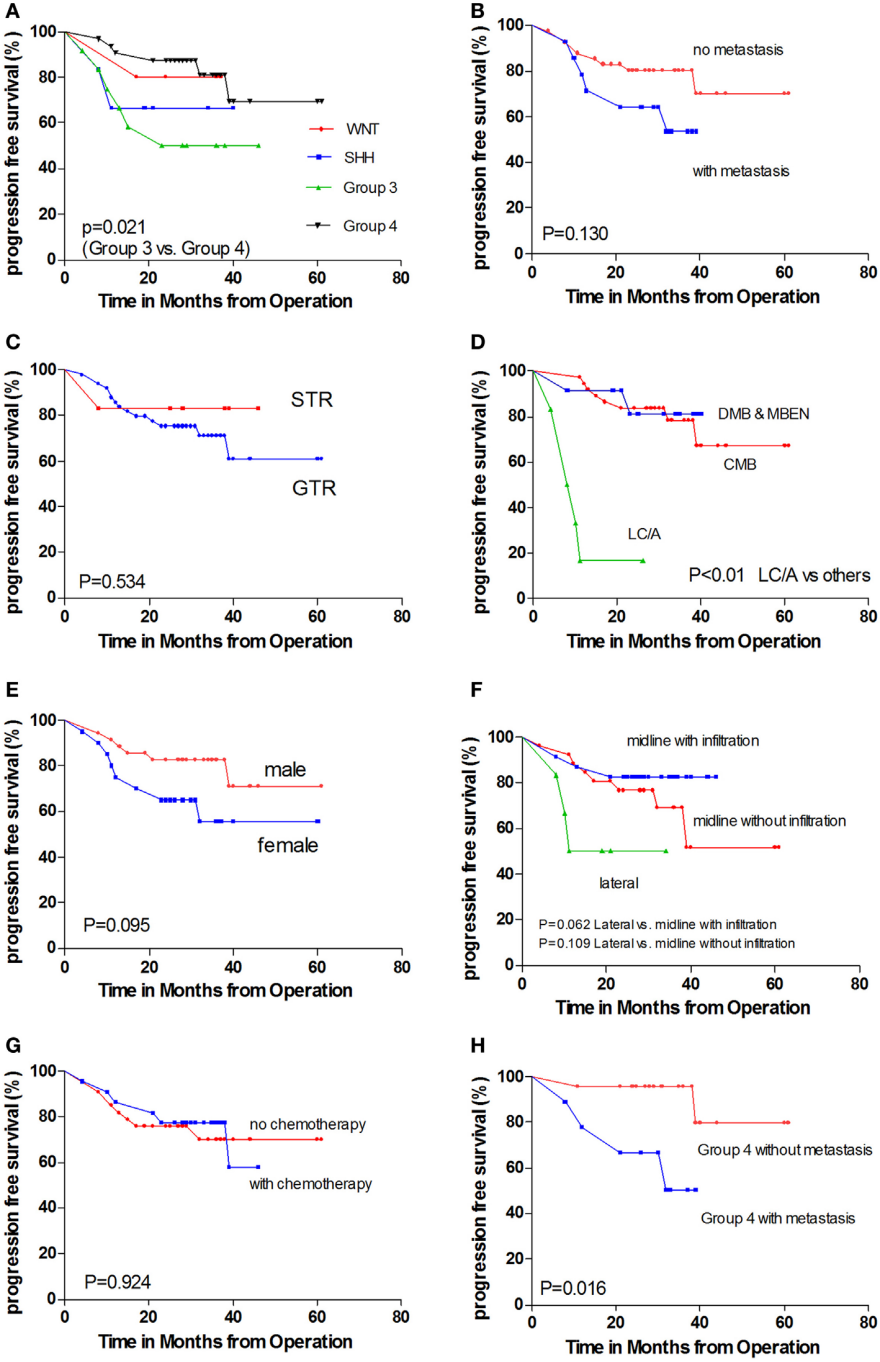
**Kaplan–Meier estimates of progression-free survival (PFS) and overall survival (OS) according to different parameters**. **(A)** Molecular subgroups. **(B)** With metastasis versus without metastasis. **(C)** GTR versus STR. **(D)** Pathology subtypes: classic histology medulloblastoma (CMB), desmoplastic/nodular medulloblastoma and medulloblastoma with extensive nodularity (DNMB and MBEN), and large cell/anaplastic (LC/A) histology. **(E)** Female patients versus male patients. **(F)** Tumor location. **(G)** With chemotherapy versus without chemotherapy. **(H)** Non-metastatic Group 4 patients versus metastatic Group 4 patients.

**Figure 4:**
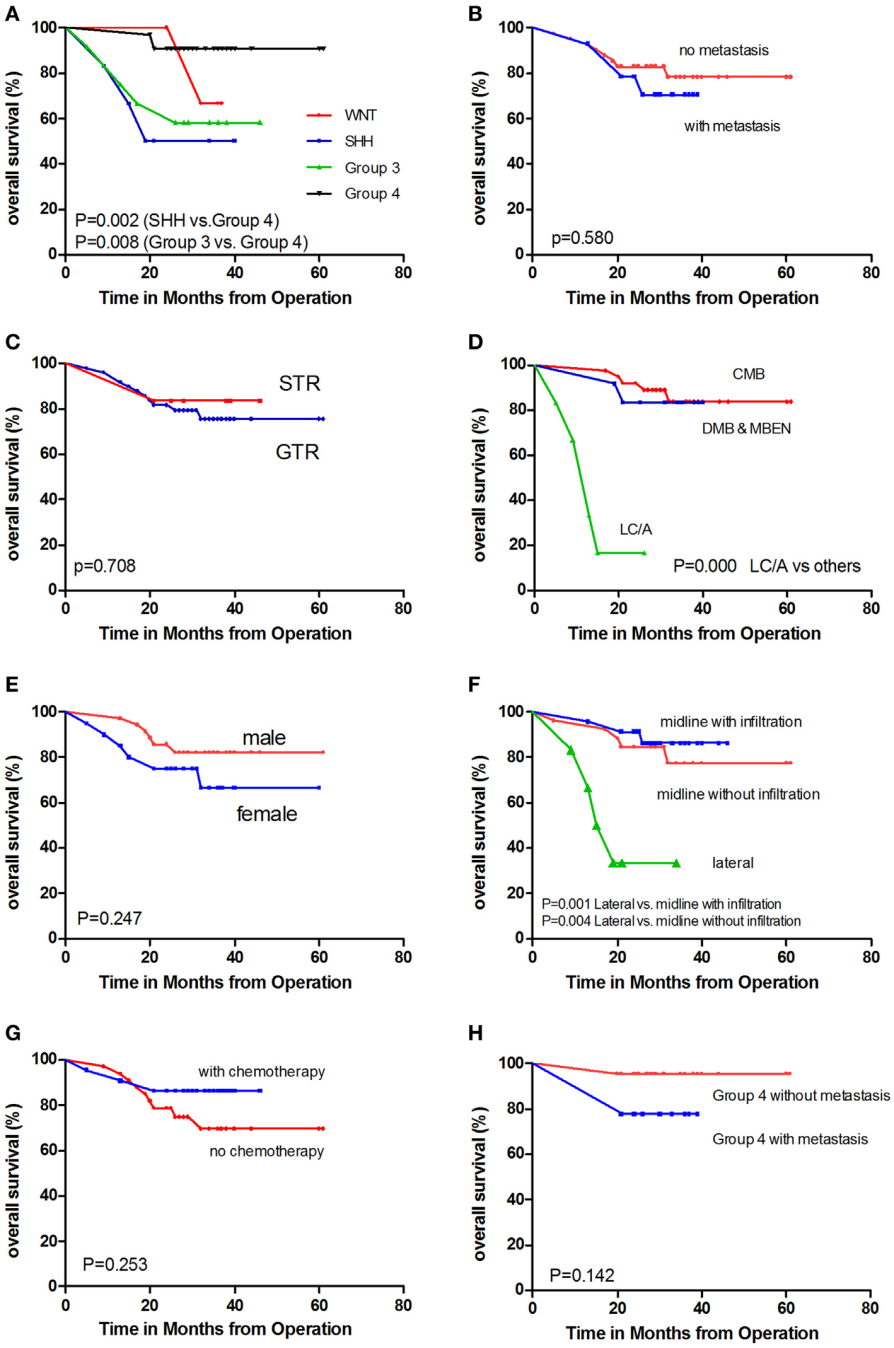
**Kaplan–Meier estimates of overall survival (OS) according to different parameters**. **(A)** Molecular subgroups. **(B)** With metastasis versus without metastasis. **(C)** GTR versus STR. **(D)** Pathology subtypes: classic histology medulloblastoma (CMB), desmoplastic/nodular medulloblastoma and medulloblastoma with extensive nodularity (DNMB and MBEN), and large cell/anaplastic (LC/A) histology. **(E)** Female patients versus male patients. **(F)** Tumor location. **(G)** With chemotherapy versus without chemotherapy. **(H)** Non-metastatic Group 4 patients versus metastatic Group 4 patients.

The 84 samples were classified into the four core subgroups by real-time RT-PCR, as described by Kunder et al. (10), using a set of 12 protein-coding genes as markers (Figure 2). The WNT subgroup exhibited concomitant overexpression of WIF1, DKK2, and MYC. In addition, the SHH subgroup displayed overexpression of HHIP, EYA1, and MYCN and underexpression of OTX2. Gene expression was difficult to differentiate between Group 3 and Group 4. Overexpression of EOMES, NPR3, MYC, and IMPG2 and reduced expression of GRM8 and UNC5D helped to distinguish Group 3 from Group 4 tumors. The PFS and OS rates for the different subgroups are displayed in Figures 3 and 4.

Among all 84 specimens, there were 9 WNT tumors, 18 SHH tumors, 20 Group 3 tumors, and 37 Group 4 tumors. A total of 55 patients who ranged in age from 4 to 18 years underwent detailed follow-up; there were 5 WNT tumors, 6 SHH tumors, 12 Group 3 tumors, and 32 Group 4 tumors. In pediatric age groups, the WNT subgroup was the smallest subgroup (5 out of 55), and group 4 was the largest (32 out of 55). In our cohort, the Group 4 and WNT subgroups had better outcomes than the SHH and Group 3 subgroups (Figures 3A and 4A). Univariate analysis revealed that Group 4 had a better PFS than Group 3 (*P* = 0.021). Furthermore, comparison of OS indicated that Group 4 had a better outcome than SHH (*P* = 0.002) and Group 3 (*P* = 0.008). No significant differences in PFS or OS were detected between the WNT subgroup and the other three subgroups.

### WNT Subgroup

One out of five patients in the WNT subgroup relapsed and ultimately died (2-year PFS, 80.0 ± 17.9%; 2-year OS, 66.7 ± 27.2%). None of the patients received maintenance chemotherapy. One patient with sellar region metastasis at diagnosis and infiltration into the V4 floor was alive at 37 months after tumor removal without progression. All tumors in this subgroup were CMB; they were all located midline, and three exhibited infiltrations into the V4 floor.

### SHH Subgroup

In the SHH subgroup, two out of six patients experienced tumor relapse, and three ultimately died (2-year PFS, 66.7 ± 19.2%; 2-year OS, 50.0 ± 00.4%). One patient was diagnosed with Gorlin syndrome and died of mediastinal lymphoma after postsurgical cerebrospinal axis irradiation (6). The tumors of two out of the six patients were classified as DNMB, those of other two patients were classified as MBEN, and those of the rest two patients were classified as AMB. The two patients with AMB both relapsed. No relapse occurred in the SHH subgroup patients with DNMB or MBEN. Furthermore, none of the patients in the SHH subgroup had metastasis at diagnosis or received maintenance chemotherapy. A total of five out of six tumors were located laterally, and one was located centrally with V4 floor infiltration.

### Group 3

Six out of 12 patients in Group 3 relapsed, and five had died at the last follow-up (2-year PFS, 50.0 ± 14.4%; 2-year OS, 66.7 ± 76.6%). Four patients had tumor metastasis at diagnosis. Only one tumor was located laterally. Three out of the five patients with V4 floor infiltration had tumor metastasis, and three out of the four patients with metastasis had V4 floor infiltration. Tumors tended to relapse earlier and in a high proportion regardless of whether metastasis had occurred. A total of four out of eight patients without metastasis and two out of four of those with metastasis relapsed no more than 24 months after tumor removal. Six patients in Group 3 received maintenance chemotherapy, which did not affect their prognoses.

### Group 4

Group 4 contained the largest number of patients and was associated with the best prognosis in our cohort. Six out of 32 patients relapsed, and 3 had died at the last follow-up (2-year PFS, 87.5 ± 5.8%; 2-year OS, 90.6 ± 5.2%). A total of 25 CMB patients, six DNMB patients, and one AMB patient were included in this subtype. Only 2 out of 23 patients without metastasis at diagnosis relapsed, and 4 out of 9 of those with metastasis relapsed. None of the Group 4 tumors were located laterally. However, seven out of nine tumors with metastasis at diagnosis had V4 floor infiltration.

A total of 9 out of 23 non-metastasized Group 4 patients received maintenance chemotherapy after radiotherapy. Only one relapse each occurred in the patients who did and did not receive chemotherapy, respectively. Chemotherapy did not have a significant effect on PFS in this subgroup (PFS, *P* = 0.685). However, it appeared to affect the PFS of the metastasized Group 4 patients. Both of the patients who did not receive chemotherapy relapsed, and only two out of the seven patients who received chemotherapy relapsed (log rank, *P* = 0.102).

### Metastasis

Tumor metastasis (M2–M3 stages) was detected in 14 out of 55 patients. A total of 41 M0-stage patients displayed a trend toward better prognosis, in addition to 14 M+ stage patients (Figures 3B and 4B; *P* = 0.130). Because postsurgical lumbar puncture for cytology examination was not routinely performed, the M1-stage patients in our cohort were classified as M0-stage patients. The number of M+ stage patients in our cohort was underestimated compared with those of the other stage patients.

No metastases were detected among the SHH subgroup patients, and only one metastasis was observed among the WNT subgroup patients. One WNT patient with metastasis was alive without progression at follow-up. In addition, high frequencies of M+-stage patients were observed in Groups 3 (4 out of 12, 33.3%) and 4 (9 out of 32, 28.1%).

### Tumor Residue

We aimed to achieve maximal tumor resection for MB patients treated at our hospital. Six patients were found to have a tumor residue of >1.5 cm^2^. Three of these patients had metastasis at diagnosis, and three did not have metastasis. Four of the patients with tumor residue received radiotherapy and maintenance chemotherapy. One out of two patients who did not receive chemotherapy experienced tumor relapse in the lumbar spine at 8 months; this patient was a metastasized Group 4 patient. No differences in tumor relapse were detected between the patients with gross total resection and those with subtotal resection (Figures 3C and 4C; *P* = 0.534).

### Pathology

Among the 55 patients, 37 had CMB, 10 had DNMB, two had MBEN, and 6 LC/A MB. Five out of six LC/A-subtype patients relapsed and had died at the last follow-up, representing a significant difference compared with the other two subtypes (Figures 3D and 4D; *P* < 0.001). The LC/A subtype was predominant in Group 3. Three out of six LC/A-subtype patients belonged to Group 3, in addition to two SHH subtype patients and one Group 4 patient.

The patients in our cohort who had DNMB or MBEN and were in the SHH subgroup did not relapse. Among the 12 patients with DNMB or MBEN histology, 0 out of 4 in the SHH subgroup relapsed, 1 out of 2 in Group 3 relapsed, and 1 out of 6 in Group 4 relapsed at the last follow-up. One of the three MBEN patients relapsed with osseous metastasis after postsurgical radiotherapy and chemotherapy. Among the 37 CMB patients, relapse occurred in 1 out of 5 WNT patients, 4 out of 6 Group 3 patients, and 5 out of 25 Group 4 patients.

### Gender

A trend toward better prognosis was observed among the male patients (Figures 3E and 4E; log rank, *P* = 0.095). The 2-year PFS rates of the male and female patients were 82.8 ± 6.4 and 65.0 ± 10.7%, respectively. Metastasis at diagnosis was more commonly observed among the male patients (7 out of 35 versus 7 out of 20). Furthermore, among the patients without metastasis, 4 out of 28 male patients relapsed and had died at the last follow-up (2-year PFS, 61.5 ± 13.5%; 2-year OS, 85.7 ± 6.6%), 5 out of 13 female patients relapsed, and four ultimately died (2-year PFS, 61.5 ± 13.5%; 2-year OS, 76.9 ± 11.7%). In addition, among the male and female patients with metastasis, three relapsed and two died.

The gender ratios of the WNT and SHH subgroups were each ~1:1, and group 3 and 4 tumors were more commonly observed in the male patients. With regard to tumor location, the gender ratio for the lateral tumors was ~1:1, and the male patients more commonly had central tumors.

### Tumor Location

A total of 26 tumors were located centrally without infiltration into the V4 floor, 23 exhibited infiltrations into the V4 floor, and 6 were located laterally. In agreement with our previous findings (5), tumor metastasis at diagnosis was more frequently observed in midline tumors with V4 floor infiltration (*P* = 0.033). Furthermore, 10 out of 23 metastases were in location subgroup 2, and only 3 out of 23 metastases and 1 out of 5 metastases were in the other two subgroups, respectively.

However, the lateral tumors were associated with worse outcomes than those in the other two locations (Figures 3F and 4F). These results differ from our previous findings. Most of the patients’ tumors (five out of six) in location subgroup 3 also belonged to the SHH subgroup. Two of the five SHH patients with lateral tumors relapsed and died after radiotherapy. One patient was diagnosed with Gorlin syndrome and developed mediastinal lymphoma at 27 months after tumor resection and radiotherapy (6). Another patient with a lateral tumor was the only one with metastasis at diagnosis, which was in location subgroup 3; this patient relapsed after 10 months.

Chemotherapy had significant effects on 33 midline tumors with V4 floor infiltration. Only 1 out of 12 patients who received chemotherapy relapsed, and none died; in comparison, 3 out of 11 of those who did not receive chemotherapy relapsed and died (OS, *P* = 0.05).

### Chemotherapy

A total of 22 patients in our cohort received maintenance chemotherapy, including 10 with and 12 without tumor metastasis at diagnosis (Figures 3G and 4G). Chemotherapy appeared to only affect the metastasized patients (*P* = 0.183) and metastasized Group 4 patients (*P* = 0.102). Among the 29 M0-stage patients who did not receive chemotherapy, 6 relapsed and 4 died; in addition, among the 13 M0-stage patients who received chemotherapy, three relapsed and died (*P* = 0.936). However, only 3 out of 10 of the M+-stage patients who received chemotherapy relapsed, compared with 3 out of 4 of those who did not receive chemotherapy (*P* = 0.183).

### Multivariate Analysis

In Cox regression analysis, comparison of the PFS rates revealed that only LC/A histology (*P* = 0.000) was an independent risk factor. In addition, comparison of the OS rates revealed that LC/A histology (*P* = 0.000) and chemotherapy (*P* = 0.050) were independent risk factors. Metastasis at diagnosis and the surgery extent did not exhibit statistical significance in our cohort. Group 3 subgroup exhibited a trend toward worse outcomes in PFS and OS.

## Discussion

To date, only one study (11) has been conducted in China using immunohistochemistry to classify pediatric patients into the WNT, SHH, and non-SHH/WNT subgroups. It subgrouped 173 tumors (median follow-up time 38.0 months; range, 0.1–118.9 months) and found that WNT tumors had a better prognosis than other two subgroups. Postoperative radiotherapy and chemotherapy were found to be prognostic factors in some patients. Another study (4) has been performed assessing adult MBs using gene expression profile and immunohistochemistry and found that Group 4 tumors had worst prognosis in adult population. The present study is the first report of prognosis of consecutive pediatric cases at a single institution in China based on four molecular classification systems. Our result is relatively consistent with previous studies (10, 12–15) regarding age distribution, high frequency of metastasis in Group 3, and male-to-female ratio of the four molecular subgroups. However, there were relatively high frequency of Group 4 tumors and low frequency of SHH tumors in present age groups.

The prognosis of the WNT subgroup patients in our cohort was not as excellent as previously described (2). None of the patients received chemotherapy, and one out of five patients relapsed and died. In our cohort, one relapsed and one metastasized patient both were 13-year-old females near the cutoff age. This result is consistent with other studies that reported that WNT tumors exhibited a secondary peak and lower OS in adolescents/young adults (16, 17). Our results suggest that chemotherapy may have an effect on this patient subgroup because it was administered at most centers reporting their better prognosis.

There is some disagreement among researchers regarding the outcomes of SHH subgroup patients (18). Previous studies have shown that infant desmoplastic and MBEN tumors, which belong to the SHH subgroup exclusively, have excellent prognosis. In addition, in Children’s Cancer Group 99703 (19), infant desmoplastic patients showed a good prognosis following treatment with marrow-ablative chemotherapy, followed by autologous hematopoietic cell rescue. Further, in HIT 2000, the survival rates were higher in patients receiving higher cumulative doses of intraventricular methotrexate (20). In our SHH subgroup, no patients received maintenance chemotherapy, which may explain the dismal prognosis. Although there were no M+ patients in our SHH subgroup, the dismal prognosis observed in this subgroup of pediatric patients strongly indicated a high risk. Kool et al. (21) have proposed that SHH-MB should be divided into three different subgroups: (1) young children with SUFU and PTCH1 mutations; (2) older children (aged 8–17 years) with germline TP53 (Li–Fraumeni syndrome) mutations and SHH pathway gene amplification; and (3) adults with PTCH1 and SMO mutations. The roles of TP53 and PTCH1 should be assessed in future clinical trials. Studies have shown that SHH-MB with a mutation downstream of SMO is resistant to SMO inhibitors (21). SHH-MB patients with a PTCH1 mutation exhibit responses to vismodegib and a longer PFS (22). Additional experimental approaches should be considered in future trial designs.

The Group 3 patients had the worst prognosis in our cohort. One-third of the patients in this subgroup had tumor metastasis at diagnosis, and half of them relapsed earlier (no more than 2 years after tumor resection). No difference in outcome was observed between the patients who received and did not receive chemotherapy. No patients in our cohort received intrathecal, intraventricular, or intensified chemotherapy. The chemotherapy dose did not reach the full dose for most patients, which may partly explain the worse prognoses compared with those reported in Western countries (2). Our result has demonstrated that stronger treatments are warranted for Group 3 patients. Consistent with previous reports, LC/A histology was enriched in Group 3. Three out of five patients with LC/A histology belonged to Group 3, and all five of the LC/A subtype patients died. LC/A histology had a significant effect on prognosis in both univariate and multivariate analyses. These results have revealed the importance of WHO histology in the molecular classification of MB. Thus, more intensified treatment is warranted for patients with LC/A histology.

The Group 4 patients in our series had the best prognosis, particularly the non-metastatic patients. The patients in our cohort received radiotherapy no more than 1 month after tumor resection. Among the non-metastatic Group 4 patients, 1 out of 13 who received maintenance chemotherapy and 1 out of 9 who did not receive chemotherapy experienced tumor relapse (*P* = 0.783). Chemotherapy did not have substantial effects in this subgroup. Some researchers have hypothesized that Group 4 patients may benefit from the timely initiation of radiation (17, 18). Our series has demonstrated that this patient subgroup has an excellent prognosis with timely radiation therapy and should be considered as a low-risk group (2). Further adjustment of the treatment strategy in this subgroup should be considered. The Group 4 subgroup remains the least characterized of all of the MB patient subgroups to date. The metastatic Group 4 patients had the worst outcomes and should be considered as high-risk patients (2). A total of four out of nine patients in our cohort experienced tumor relapse, and two out of nine patients died. However, chemotherapy may be helpful for the treatment of metastasized Group 4 patients, as only two out of seven of those who received chemotherapy relapsed in this study. In addition, most relapses in the Group 4 patients were disseminated metastases (five out of six), consistent with previous reports (23).

In our study, the non-metastatic Group 4 patients had excellent outcomes, including most of the patients who only received radiotherapy. The pediatric patients in the SHH subgroup had dismal outcomes, and no M+-stage SHH patients were identified in our cohort. Chemotherapy did not significantly affect the non-metastatic Group 4 patients. However, this study was limited by the small number of patients and short follow-up period. It should be noted that Group 4 tumors recur later than tumors in the other subgroups (23), and the median follow-up time of the Group 4 patients in our series was only 30.5 months. Another shortcoming of the present study was the lack of status of CTNNB1, MYC, TP53, etc., due to funding limitations. Further prospective international studies should be performed in China.

## Conclusion

In our cohort of MB patients aged between 3 and 18 years, the non-metastasized Group 4 patients had an excellent prognosis. Maintenance chemotherapy did not significantly affect these patients. The SHH subgroup and Group 3 patients had worst prognoses; thus, these patients should receive more intensive treatment. LC/A histology had a dismal prognosis in our cohorts, which warrants intensive treatment.

## Author Contributions

TJ and CL conceived and led the project. YW and JW performed the 12-gene signature assay for gene expression-based subgrouping. JD, YZ, and Raynald supervised and performed quality control for the subgrouping. XQ, TJ, YW, and JW acquired the data (acquired and managed patients, selected and characterized samples, provided disease-specific histopathological analysis). JW and JD provided WHO histopathological analysis. TJ and YW co-wrote the manuscript with input from all co-authors.

## Conflict of Interest Statement

The authors declare that the research was conducted in the absence of any commercial or financial relationships that could be construed as a potential conflict of interest.
