# Supplementary material for: A Retrospective Study of Progression-Free and Overall Survival in Pediatric Medulloblastoma Based on Molecular Subgroup Classification: A Single-Institution Experience
Source: Front Neurol. 2017 May 12;8:198. doi: 10.3389/fneur.2017.00198 (PMC5427081; doi:10.3389/fneur.2017.00198)
Supplement: Supplementary file 1 [file Table_1.docx]

**Supplementary Table 1: Nucleotide sequences of the primers used for Real -time RT- PCR analysis.**

| **Gene Name** | **Nucleotide Sequence (5' to 3')** |
| --- | --- |
| *WIF1-F* | GCAGCACACGCCTTCACTTA |
| *WIF1-R* | GTCGGAGTTCACCAGATGTAATTG |
| *DKK2-F* | ACCAGGGGATGCACAGTCTA |
| *DKK2-R* | TACGGACACAGGACCTCACA |
| *EYA1-F* | CGTGTCTGCATTTTCTTTCGATA |
| *EYA1-R* | ACGCACATAGGGAGTAATGCAA |
| *MYCN-F* | CATAAGGGGTTTGCCATTTG |
| *MYCN-R* | CCACCTCTCATTACCCAGGA |
| *HHIP-F* | GAATGCAGAGCCACGGTACA |
| *HHIP-R* | GGTGCAGTAGCCGTTTCGA |
| *OTX2-F* | TTGCAAAGTGATCAAAAGCATTC |
| *OTX2-R* | CCTCCCTTCCTTCACAACTTAGTTT |
| *EOMES-F* | GAGTGTTAAGGTGTTTTGCTTCTGAA |
| *EOMES-R* | GGCAGAATGTAACAGCAAAATGTC |
| *NPR3-F* | GGTCCTGGGAATCTGAGCTTT |
| *NPR3-R* | CTGTCACAAGGTCTATCCCATGTC |
| *IMPG2-F* | GACGGTCACTATTCTCAAATCCATT |
| *IMPG2-R* | TTCCAGGCAATTCTTTACATCATG |
| *GRM8-F* | TCCAGCTCCTTGGAGTGTTTG |
| *GRM8-R* | CCGCTGCTCTCCATAGTCAAT |
| *UNC5D-F* | GGTCACAGGAATCGTGAAGGA |
| *UNC5D-R* | ATGACAATGGGCCACAGACA |
| *MYC-F* | AAATGTCCTGAGCAATCACCTATG |
| *MYC-R* | TTATGCCCAAAGTCCAATTTGA |
| *GAPDH-F* | TGACTTCAACAGCGACACCCA |
| *GAPDH-R* | CACCCTGTTGCTGTAGCCAAA |

F and R stand for Forward and Reverse primer respectively.
